# Human Papillomavirus in Head and Neck Squamous Cell Carcinoma of Unknown Primary Is a Common Event and a Strong Predictor of Survival

**DOI:** 10.1371/journal.pone.0110456

**Published:** 2014-11-04

**Authors:** David Hebbelstrup Jensen, Nora Hedback, Lena Specht, Estrid Høgdall, Elo Andersen, Marianne Hamilton Therkildsen, Lennart Friis-Hansen, Bodil Norrild, Christian von Buchwald

**Affiliations:** 1 Department of Otorhinolaryngology, Head and Neck Surgery and Audiology, Rigshospitalet, University of Copenhagen, Copenhagen, Denmark; 2 Department of Oncology, Rigshospitalet, University of Copenhagen, Copenhagen, Denmark; 3 Department of Pathology, Herlev Hospital, University of Copenhagen, Herlev, Denmark; 4 Department of Oncology, Herlev Hospital, University of Copenhagen, Herlev, Denmark; 5 Department of Pathology, Rigshospitalet, University of Copenhagen, Copenhagen, Denmark; 6 Centre for Genomic Medicine, Rigshospitalet, University of Copenhagen, Copenhagen, Denmark; 7 Department of Cellular and Molecular Medicine, The Panum Institute, University of Copenhagen, Copenhagen, Denmark; Peking University Cancer Hospital and Institute, China

## Abstract

**Background:**

The purpose of this study was to examine the prevalence of human papillomavirus (HPV) in patients with head and neck squamous cell carcinoma of unknown primary (CUP).

**Methods:**

All patients diagnosed with and treated for CUP between January 1, 2000, and June 1, 2011, at two Danish medical centers were included. All patients received a thorough diagnostic work-up, including FDG-PET, before being diagnosed as CUP. We determined the HPV status in all patients using a combination of HPV DNA PCR and p16 stain. In addition, clinical information on the study patients was retrieved from clinical records.

**Results:**

Of the identified 60 patients with CUP, 13 were shown to be positive for HPV DNA, amounting to 22% of the study population. In addition, we were able to show a clear disease-free and overall-survival benefit in the HPV-positive group, with a hazard ratio of 0.16 (95% CI: 0.038–0.67) for over-all survival. This survival benefit was also apparent when adjusted for advanced age in a multivariate Cox regression analysis.

**Conclusion:**

A fairly large percentage of CUP cases are HPV-related, and because this is related to both the location and prognosis, we recommend HPV testing as part of the diagnostic work-up.

## Introduction

Not uncommonly, head and neck squamous cell carcinoma (HNSCC) presents as a non-tender mass in the neck, i.e., a lymph node metastasis to the neck. In a small percentage of the total cases, it is impossible to ascertain the location of the *primary* tumor after extensive work-up, and the case is then defined as a cancer of unknown primary (CUP). The incidence of CUP of the head and neck is estimated to be approximately 2% of all HNSCC cases [1], and primarily affects men in the 55–60 year age group with a significant smoking and/or drinking history. When squamous CUP is suspected, it is recommended to perform a fine needle aspiration of the lymph node c.f. Danish Head and Neck Cancer Association (DAHANCA) guidelines [2]. When the fine needle aspiration shows carcinoma cells, an extensive work-up is performed, including physical examination, endoscopy, CT and/or MR and PET followed by tonsillectomy and base of tongue biopsies, to find the primary tumor site. In the majority of cases, the work-up can identify the primary tumor; however, in a subset of patients, the primary tumor cannot be located. This study examines patients in which the primary tumor was never found. The pathological diagnosis will often reveal a squamous cell carcinoma (75%) [3].

In several retrospective analyses [4], human papillomavirus (HPV) has been found to be the most important biomarker for disease-free survival and overall survival (OS) in patients with oropharyngeal cancer. HPV-associated oropharyngeal cancer has been present for many years but an increasing incidence in the last 20 years have been observed [4]. As such, an increased incidence of HPV-related CUP would therefore be expected. This is particularly relevant to consider because it has been observed that HPV-positive cancers are prone to early metastasis, i.e., are diagnosed at an advanced N-stage with a small T-site [5]. Due to the favorable prognosis of HPV-related oropharyngeal cancers, treatment deintensification of HPV-associated oropharyngeal cancer is now being explored in clinical trials (NCT01084083 Eastern Cooperative Oncology Group 1308 and NCT01302834 Radiation Therapy Oncology Group 1016).

The standard pathological work-up of carcinomatous lymph node metastases in the neck from an unknown primary tumor does not in all centers include a strict work-up to ascertain HPV status, although some guidelines recommend this [6]. Nevertheless, the HPV status of the lymph nodes could potentially contribute to both stratifying the patients with regard to treatment and also to localizing the primary tumor because the vast majority of HPV-positive HNSCCs arise from the oropharynx [7], [8]. The recommendations of treating these patients with “true” primary unknown cancer generally include radiation therapy involving large volumes to ensure the inclusion of the suspected primary tumor. This leads to increased morbidity because it is not possible to narrow down the irradiated volume to include only the relevant primary tumor area. If the cancer was shown to be HPV-positive, this could potentially enable the radiation oncologist to narrow the field to only include the oropharynx and any additional regional metastases present. Another possibility is to perform transoral robotic-assisted surgery in a selected number of patients to help locate the primary tumor [9]–[11].

We therefore sought to determine the prevalence of HPV in the metastases of patients with neck node squamous cell carcinoma from an unknown primary referred to and treated as CUP, after having been through an extensive diagnostic work-up, at two Danish medical centers between the 1^st^ of January 2000 and the 1^st^ of June 2011. Patients referred with the diagnosis “metastatic cervical lymph nodes with unknown primary” were examined to obtain the HPV prevalence and to examine whether their HPV status had any influence on their chances of long-term survival. To our knowledge, no other studies have been able to show that patients with HPV-related CUP have a survival benefit compared to non-HPV-related CUP, which could be due to a lack of power [8], [12]–[20]. We chose to use a modified version of the algorithm proposed by Smeets et al. to define HPV-positive samples [21]. Here we report that HPV is a strong prognostic factor for both over-all and progression-free survival in patients diagnosed and treated as CUP.

## Material and Methods

### Patients

Formalin-fixed, paraffin-embedded (FFPE) biopsies from patients referred to Herlev University Hospital and Rigshospitalet, Copenhagen University Hospital, between the 1^st^ of January 2000 and the 1^st^ June 2011 with the diagnosis carcinoma lymph node metastasis from an unknown primary tumor were included, totaling 105 patients from Rigshospitalet and 130 patients from Herlev University Hospital. Before being given the diagnosis cancer of unknown primary *all* patients had to have undergone a complete diagnostic work-up, including FDG-PET, random biopsies and endoscopic examinations in general anesthesia. In addition only patients in whom FFPE biopsy material on squamous cell carcinoma was present and who were treated with irradiation with an intention to cure were included in the study, leaving a total of 60 patients for the analyses. Histological diagnosis of the original metastasis was based on the examination of hematoxylin-eosin (H&E) stained tissue by an expert pathologist according to the modified WHO classification of tumors [22]. This study was approved by the Scientific Ethics Committee of the Capital Region of Denmark (registration number H-C-2008-080). Since a large group of patients had died or were severely ill, it was judged by the ethical committee that it would cause more distress than good to be informed about the project; it was therefore judged not to be necessary to obtain informed consent. Patient records were anonymized and de-identified prior to analysis.

### DNA extraction

All original tumor slides were evaluated by an expert pathologist for the presence of squamous cell carcinoma in the specific tumor block. From the selected tumor blocks, 10 µm-thick sections were cut under clean conditions on a microtome. Fixative removal was performed in xylene and absolute ethanol washes (two times). Pellets were dried and resuspended in 180 µl ATL buffer (QIAmp DNA Mini Tissue Kit, Qiagen Denmark, Copenhagen) and 5 µl proteinase K and digested overnight at 56°C. After digestion, the samples were set up in the automated sample preparation system Qiagen QIAcube (Qiagen Denmark, Copenhagen) using the Qiagen QIAamp DNA mini kit (Qiagen Denmark, Copenhagen) according to the manufacturer's specifications, with a final elution volume of 200 µl. The purified DNA was subsequently quantified using a NanoDrop (ND-1000) spectrophotometer (NanoDrop Technologies, Wilmington, DE, USA).

### Human papilloma virus polymerase chain reaction and p16 IHC

Polymerase chain reaction for HPV was performed using consensus primers GP5+/GP6+ [23]. Validation of the DNA quality and the efficacy of the PCR reaction were carried out using the housekeeping gene GAPDH. Immunohistochemistry for p16 was carried out using the Ventana Benchmark Ultra autostainer with the optiView detection kit and the p16 monoclonal antibody E6H4 (Roche). The slides were evaluated as positive if there was a strong and diffuse nuclear and cytoplasmic reaction in more than 70% of the tumor cells [24]. In addition, a confirmatory slide was stained with H&E to evaluate the presence or absence of tumor tissue in the p16-stained sections.

### Statistical analysis

The clinical and pathological data were analyzed by Fisher's exact test for categorical variables and Student's t-test for continuous variables. A p-value of less than 0.05 was considered statistically significant. Kaplan-Meier estimates and Cox regression were used to estimate the factors influencing overall survival. To test for correlation, we used Pearson's correlation coefficient. All tests were carried out using SPSS version 19. We divided the patients into the following categories according to their status at the last known follow-up: 1) no sign of recurrence at any time during follow-up, 2) treated for recurrence with no signs of current recurrence, 3) recurrence in spite of curatively intended treatment, 4) never recurrence-free. Progression-free survival was defined as the time to recurrence, and patients were censored when they experienced a recurrence or if they were never recurrence-free. To study overall survival, we divided the patients into the following categories at the last known follow-up: 1) alive, 2) died of another cancer type, 3) died due to CUP, 4) died of other causes. Age was a continuous variable in our survival analysis. To comply with the open access policy all data have been made available as Table S1.

## Results

Of all the patients referred in the 10-year period, 60 patients met the inclusion criteria. The study population was included from the 1^st^ of January 2000 to the 1^st^ of June 2011, with a median follow-up time of 3.0 years (95% CI: 1.8–5.3). Only patients in whom 1) the primary diagnosis was of a squamous cell carcinoma exclusively in the neck lymph nodes and 2) the primary tumor could not be found in the diagnostic work-up were included. The clinical characteristics of the patients are shown in Table 1. Of the 60 patients, a complete medical history for smoking and alcohol could be obtained for 52, whereas overall survival and progressions-free survival could be obtained for every patient. All patients were treated with curatively intended radiotherapy, without chemotherapy or hypoxic radiosensitizer, according to national guidelines [2].

**Table 1:** Clinical and pathological characteristics in relation to overall and progression-free survival.

		Overall survival			Progression-free survival		
		Hazard ratio	95% CI	P	Hazard ratio	95% CI	P
**n**	60				60		
**Gender (m/f)**	46/14	0.64	0.29–1.4	0.25	0.65	0.29–1.4	0.28
**Age (years)**	N.A.	1.04	1.001–1.089	0.047*	1.0	0.97–1.0	0.77
**Smoking (high/low)**	37/15	1.3	0.55–2.8	0.59	0.76	0.34–1.7	0.50
**Alcohol consumption (high/low)**	26/26	1.3	0.64–2.8	0.44	1.0	0.5–2.4	0.83
**Recurrence (no recurrence/recurrence or never tumor free)**	31/29	11	4.4–28	<0.001	N.A	N.A	N.A.
**HPV+**	13/47	0.16	0.038–0.67	0.012*	0.30	0.09–0.98	0.05
**P16+**	13/47	0.071	0.010–0.52	0.009*	0.18	0.04–0.77	0.02*
**Both HPV+ and p16+**	11/49	0.091	0.012–0.68	0.018*	0.23	0.05–0.97	0.04*
***Differentiation grade***							
Well or moderately differentiated	26	1			1		
Poorly differentiated	13	0.94	0.42–2.1	0.88	1.1	0.48–2.7	0.75
Other	21	0.31	0.12–0.79	0.014*	0.52	0.21–1.3	0.16
**Non-keratinizing morphology (Yes/no)**	18/42	0.23	0.082–0.67	0.007*	0.39	0.15–1.0	0.054
**Cystic morphology (yes/no)**	20/40	0.61	0.28–1.4	0.23			

Footnote: *, significantly associated with survival, N.A, not applicable.

### Prevalence of human papilloma virus DNA and p16 overexpression in metastatic lymph nodes

Of the 60 patients in our study group, 13 (22%) were positive by HPV PCR, and 13 were p16 positive (22%). When combining p16 and HPV PCR, 11 were positive by both markers (18%). Overall, there was a significant correlation between a positive HPV status and a lower alcohol consumption and shorter smoking history when using either p16, HPV or both as a marker of HPV infection (Table 2). Additionally, there was a significant correlation between gender and HPV, with the HPV-positive cancer patients more likely to be male. In fact, only one female patient tested positive for HPV by PCR, but was negative for p16. We did not find a correlation between age and the presence of HPV by any method (Table 2). In addition, we did not observe a rising incidence in HPV- or p16-positive cancers when comparing the incidence in 2000–2005 to 2005–2011 (Table 2). Also we did not observe a significantly rising incidence in CUP in the same period, from 28 to 32 (Table 2). Moreover, we observed a significant correlation between patients having a lower risk of recurrence and HPV status (Table 2). In fact, none of the patients with an HPV-positive cancer were in the group of patients who did not respond to treatment, i.e., no recurrence-free survival (data not shown).

**Table 2:** Correlations between patient characteristics and HPV and p16 status.

	n	HPV+	r	P	P16+	r	P	HPV+ and p16+	r	P
**Smoking**										
High	37	5	−0.29	0.034*	5	−0.23	0.10	4	−0.27	0.052
Low	15	6			5			5		
**Alcohol consumption**										
High	26	1	−0.42	0.002*	2	−0.29	0.035*	1	−0.36	0.010*
Low	26	10			8			8		
**Age**										
High (>55)	41	7	−0.16	0.20	8	−0.08	0.55	6	−0.14	0.27
Low (<55)	19	6			5			5		
**Gender**										
Male	46	12	0.194	0.13	13	0.29	0.025*	11	0.26	0.043*
Female	14	1			0			0		
**Period**										
Before 2005	28	6	0.08	0.57	6	0.13	0.97	6	0.12	0.57
After 2005	32	7			7			5		
**Morphology**										
Non-keratinizing	18	12	0.72	<0.001*	12	0.72	<0.001*	11	0.72	<0.001*
Other	42	1			1			0		
**Morphology**										
Cystic	20	10	0.49	<0.001*	11	0.57	<0.001*	9	0.49	<0.001*
Non-cystic	40	3			2			2		
										
**Recurrence**										
No recurrence	31	10	−0.12	0.040*	11	−0.35	0.007*	9	−0.29	0.027*
Recurrence or never tumor free	29	3			2			2		

Footnote: * significant correlation. HPV, p16 or HPV and p16 status is compared to the patient characteristics in the left. P values are from a Pearson's correlation and applies to each test.

### Pathological characteristics

An expert in head and neck pathology scored the following parameters on the lymph node metastases: differentiation grade, presence of cysts and non-keratinizing morphology. The differentiation grade was divided into well, moderate and poorly. In addition, we observed some patients with a hybrid morphology, and along with the non-keratinizing, they are grouped into the category “other” in Table 2. Of the 60 patients, 18 had a non-keratinizing morphology, with a strong correlation to both HPV and p16 status (P<0.001, Table 2). We also observed a strong correlation between cystic metastases and HPV and p16 status (P<0.001, Table 2).

### Overall survival and progression-free survival

**Figure 1:**
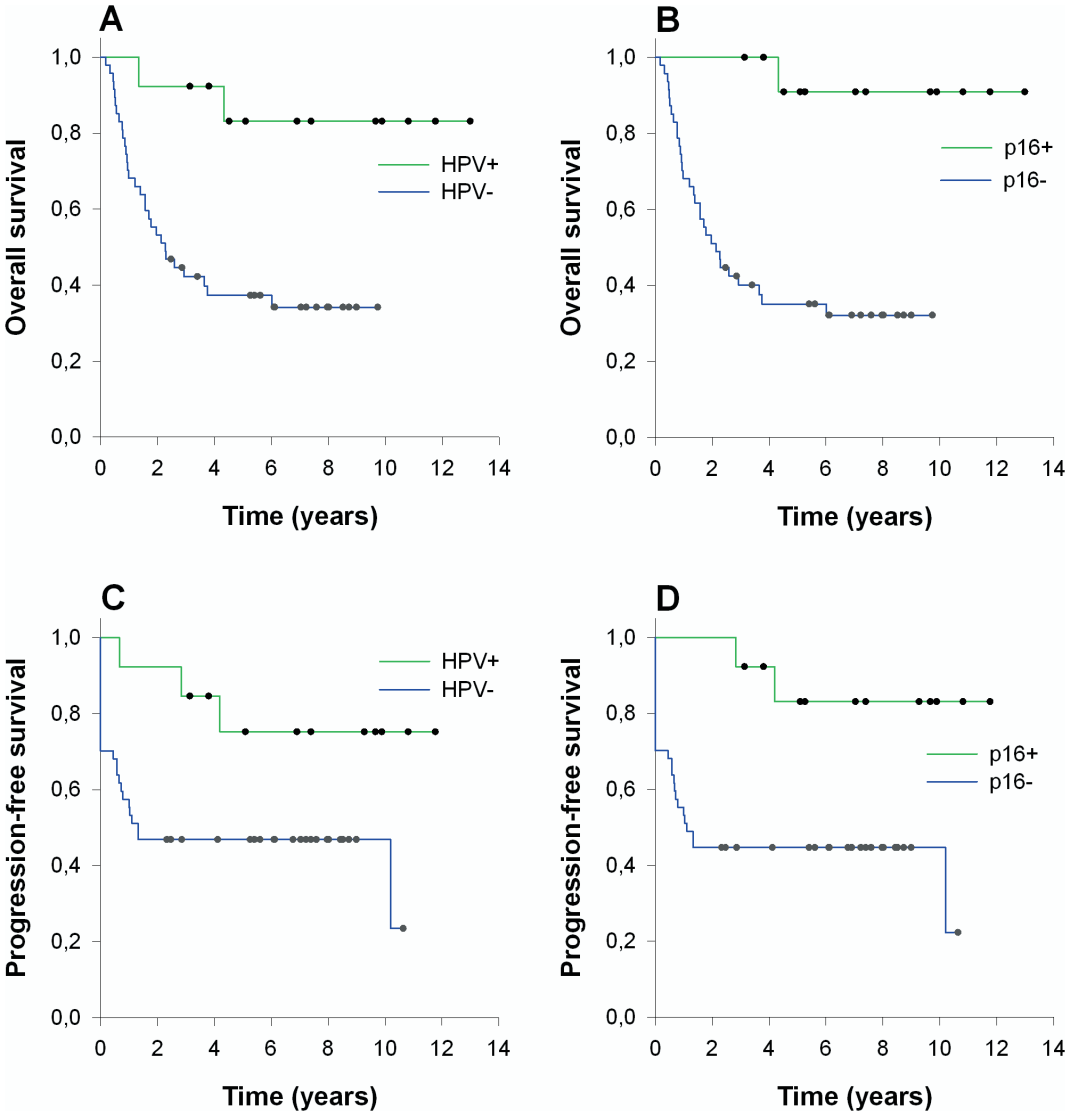
Survival based on HPV status in patients with unknown primary. Kaplan-Meier curves on overall survival (A and B) and progression-free survival (C and D), as stratified based on HPV and p16 staining. The survival differences were all statistically significant, as based on logrank tests (P<0.05).

Of the 60 patients, 32 died within a median of 16 months (95% CI: 10–24), and 15 developed a recurrence within a median of 12 months (95% CI: 8–16) in the head and neck region. A total of 14 patients never experienced progression-free survival. Of the 15 patients with a recurrence, 3 patients were treated for a recurrence and were recurrence-free at the last follow-up. There was a significantly longer progression-free survival in the HPV-positive group compared to the HPV-negative group (Table 1, Fig. 1). In addition, there was a significant association between HPV and response to treatment. The HPV-positive group was more likely to be responsive to radiotherapy, i.e., becoming disease free after treatment, and 2 out of the 3 with a treatable recurrence were HPV positive.

Overall survival in our cohort was clearly related to the presence of HPV, as shown in Fig. 1 and Table 1. This was observed as a hazard ratio of 0.16 (95% CI: 0.038–0.67) for those positive for HPV compared to the HPV-negative patients (P<0.05). This survival benefit was also found when using p16 alone or in combination with HPV (Table 1). In addition, age was found to mildly influence overall survival, with a hazard ratio of 1.04 (95% CI 1.001–1.089). There was a significant negative correlation between the development of a recurrence and HPV status (Table 2). We also performed a multivariate cox regression analysis (Table 3) with all the factors that were shown to significantly influence over-all survival from the univariate analysis (Table 1). Also, smoking and alcohol history was tested as significant covariates due to their correlation to HPV status, although not shown to significantly influence over-all survival. Afterwards we eliminated factors from the model, one by one, until only significant factors remained, i.e. backwards elimination. Due to the fact that p16 and HPV were highly correlated and both were correlated to recurrence, only p16 were remained as an independently prognostic factor, since p16 status slightly outperformed HPV status in our multivariate analysis. HPV and recurrence status were not independently related to overall survival. After backwards elimination, only the p16 status and age remained as independently prognostic factors, with hazard ratios of 0.071 (95% CI: 0.010–0.52) and 1.04 (95% CI: 1.00–1.09), respectively (P<0.05). Of note, neither overall nor progression-free survival was related to a longer smoking or drinking history.

**Table 3:** Multivariate cox regression analysis of over-all survival (final model).

	Hazard ratio	95% CI	P
p16	0.71	0.010–0.52	0.009
Age	1.004	1.001–1.090	0.047

## Discussion

The treatment and diagnostic work-up of patients with CUP of the head and neck represent a challenge for physicians. This is, to the best of our knowledge, the first study to show a marked survival benefit among patients with HPV-positive as opposed to HPV-negative CUP of the head and neck. Several studies have examined the usefulness of HPV as a marker to identify the primary tumor [12], [16], [19], [25], but in the strictest sense do not describe “true” CUP because in all these studies, the primary tumor was eventually found, thus excluding the possibility of true CUP representing a distinct disease entity. In our cohort, patients with HPV-positive tumors had a markedly better survival than did HPV-negative patients. The favorable outcome was related to the fact that the HPV-positive cancers responded better to treatment: all the HPV-positive patients had a response to treatment, and only one of the patients developed a recurrence that could not be managed. In addition, this marked survival benefit was observed not to be related to their shorter smoking and drinking history, or advancing age, as could be seen from the multivariate analysis.

In our patient group, the percentage with an HPV-positive cancer was 22%, which is in agreement with a study from the USA, which found an HPV prevalence in CUP of 28% using FISH [19]. However studies by Tribius and Sivars examined CUP patients for HPV, and found a higher HPV-prevalence of 37 and 40%, respectively [26], [27]. However, in the study by Sivars the patient group involved patients with a primary but not a final diagnosis of CUP, thus not representing the same patient category as the one is this study, with only diagnosed CUP patients after a FDG-PET examination, i.e. “true” CUP. In addition, we wanted to examine whether there was a rising incidence in HPV-positive CUP patients in our study period. We therefore divided our study period into two, one before and one after 2005, because an increasing incidence has been observed in oropharyngeal cancers in Scandinavia since 2000 [28]. We were however not able to show an increasing incidence in CUP or in HPV positive CUP. This could be related to many factors, such as a change in the diagnostic work-up during the period [29] or lack of power due to the relatively few patients (n = 60). One limitation of our study is that we were only able to examine metastases; these may have a different biology from the primary tumor, though studies have shown a high correlation between HPV DNA in primary tumors and their metastases [30].

HPV-related oropharyngeal cancers have in many studies been shown to be related to a different patient group than the well-known head and neck cancer patients, in that the patients with HPV-related oropharyngeal cancers are younger and generally do not have a long smoking and/or drinking history. In our cohort, we show a tendency in the patient characteristics, whereby HPV-related CUP was more likely found in males with a lower intake of alcohol and a shorter smoking history.

In addition to its role as a *prognostic* biomarker, the presence of HPV in CUP could also be valuable for finding the primary tumor, as has previously been suggested [8], [12], [25], [31]. However, to date, no prognostic study using HPV status to help locate the primary tumor has been performed [32]. Nevertheless, in small case-series [8], [12]–[20], the prevalence of HPV in true CUP has been studied (in some studies, only p16 overexpression was examined). All have a very small sample size, and the prevalence is therefore subject to small sample size effects, as demonstrated by the variation in HPV-positive CUP from 0 out of 1 (0%) [12] to 2 out of 2 (100%) [17].

We recommend that the presence or absence of HPV/p16 should be determined early in the diagnostic work-up of CUP patients because their status may influence treatment decisions. Because HPV-related cancers have a better prognosis due to radiotherapy and surgery, the presence or absence of HPV may become an important factors in deciding the best treatment plan, and clinical trials testing de-escalation therapy in HPV-positive oropharyngeal cancers have been initiated.

Knowledge about the absence or presence of HPV may prove useful while performing panendoscopy, as patients with a HPV-positive tumor would be expected to benefit more from tonsillectomy and/or transoral robotic resection of the upper-most layer of the tongue tonsil, to find the primary tumor [9]–[11]. Additionally, the HPV-negative tumors would be expected to benefit less from tonsillectomy and multiple base of tongue biopsies because a lower success rate in finding the primary tumor would be expected; thus, the risks and benefits should be taken into account in each case. We also acknowledge that the use of random biopsies for the identification of the primary tumor is somewhat controversial [33]. Regardless, patients with a HPV-negative tumor should be considered for a more aggressive treatment plan than the HPV-positive patients, because this patient group had a markedly lower overall survival. Chemotherapy should be also considered for patients with other clinical characteristics generally known to be associated with a poorer outcome, including extracapsular spread and multiple involved lymph nodes.

For unknown primary in the head and neck, historically, radiotherapy plans included bilateral fields in the neck, which included the nasopharynx, oropharynx, larynx and hypopharynx. These fields are highly likely to lead to unnecessary toxicity because the most likely origin of a HPV-positive unknown primary is situated in the oropharynx [31]. Therefore some recommend removing the larynx and hypoharynx from the field. In addition, it has been shown that patients with CUP treated with ipsilateral radiotherapy compared to a more comprehensive radiotherapy did not demonstrate a worse locoregional control or overall survival [34]. The best radiotherapy plan for patients with CUP is however not universally agreed upon [35]. Whether treatment can be modified according to HPV-status will need to be tested in prospective trials.

Another support in the diagnosis of an HPV-positive cancer is the use of tumor morphology [36]. In our dataset, when the tumor was non-keratinizing, the chance of being positive by HPV PCR was 67%, which however is not sufficient for the diagnosis of an HPV-positive cancer. A non-keratinizing morphology should give rise to a strong suspicion as to a tonsillar or base of tongue location [37]. We also advocate that a dedicated head and neck pathologist reviews the tumor because what is routinely perceived as a poorly differentiated tumor may actually be merely a non-keratinizing HPV-positive carcinoma [38]. We also confirmed a strong relationship between cystic metastases and HPV, as has previously been observed [17].

In approximately 2% of patients with a squamous cell carcinoma of the neck, the primary tumor will not be located, even after completing a comprehensive work-up. It could be speculated that improved tumor imaging could advance our ability to find the primary tumor. However for the time being, a small subset of primary tumors cannot be found, and in these patients HPV testing may be valuable; both in determining the location, but also in prognostication and in the future possibly also treatment planning.

## Supporting Information

Table S1 Raw data for all pathological and clinical characteristics pertaining to this study, to comply with the open access policy.
(XLSX)

## References

[1] Grau C, Johansen LV, Jakobsen J, Geertsen P, Andersen E, et al (2000) Cervical lymph node metastases from unknown primary tumours: Results from a national survey by the Danish Society for Head and Neck Oncology. Radiotherapy and Oncology 55: 121–129. 10799723 10.1016/s0167-8140(00)00172-9

[2] DAHANCA Website (Danish Head and Neck Cancer Group) Database registering of head and neck cancer in Denmark. Available: http://www.dahanca.dk. Accessed 2013 Jun 1.

[3] Califano J, Westra WH, Koch W, Meininger G, Reed A, et al (1999) Unknown Primary Head and Neck Squamous Cell Carcinoma: Molecular Identification of the Site of Origin. Journal of the National Cancer Institute 91: 599–604. 10203278 10.1093/jnci/91.7.599

[4] Marur S, D'Souza G, Westra WH, Forastiere AA (2010) HPV-associated head and neck cancer: a virus-related cancer epidemic. The Lancet Oncology 11: 781–789. 20451455 10.1016/S1470-2045(10)70017-6 PMC5242182

[5] Hafkamp HtC, Manni JJ, Haesevoets A, Voogd AC, Schepers M, et al (2008) Marked differences in survival rate between smokers and nonsmokers with HPV 16-associated tonsillar carcinomas. International Journal of Cancer 122: 2656–2664. 18360824 10.1002/ijc.23458

[6] DSG THaNC Routine HPV testing in Head and Neck Squamous Cell Carcinoma. Available: https://www.cancercare.on.ca/common/pages/UserFile.aspx?fileId=279836. Accessed 2014 May 7.

[7] El-Mofty S, Zhang M, Davila R (2008) Histologic Identification of Human Papillomavirus (HPV)-Related Squamous Cell Carcinoma in Cervical Lymph Nodes: A Reliable Predictor of the Site of an Occult Head and Neck Primary Carcinoma. Head and Neck Pathology 2: 163–168. 20614311 10.1007/s12105-008-0066-1 PMC2807564

[8] Begum S, Gillison ML, Ansari-Lari MA, Shah K, Westra WH (2003) Detection of Human Papillomavirus in Cervical Lymph Nodes: A Highly Effective Strategy for Localizing Site of Tumor Origin. Clinical Cancer Research 9: 6469–6475. 14695150

[9] Mehta V, Johnson P, Tassler A, Kim S, Ferris RL, et al (2013) A new paradigm for the diagnosis and management of unknown primary tumors of the head and neck: A role for transoral robotic surgery. The Laryngoscope 123: 146–151. 23154813 10.1002/lary.23562

[10] Patel SA, Magnuson JS, Holsinger FC, Karni RJ, Richmon JD, et al (2013) Robotic Surgery for Primary Head and Neck Squamous Cell Carcinoma of Unknown Site. JAMA 139: 1203–1211. 10.1001/jamaoto.2013.5189 24136446

[11] White HN, Moore EJ, Rosenthal EL, Carroll WR, Olsen KD, et al (2010) Transoral robotic-assisted surgery for head and neck squamous cell carcinoma: one- and 2-year survival analysis. 136: 1248–1252. 10.1001/archoto.2010.216 21173375

[12] Weiss D, Koopmann M, Rudack C (2011) Prevalence and impact on clinicopathological characteristics of human papillomavirus-16 DNA in cervical lymph node metastases of head and neck squamous cell carcinoma. Head & Neck 33: 856–862. 20737490 10.1002/hed.21548

[13] Armas GL, Su C-Y, Huang C-C, Fang F-M, Chen C-M, et al (2008) The impact of virus in N3 node dissection for head and neck cancer. European Archives of Oto-Rhino-Laryngology 265: 1379–1384. 18421466 10.1007/s00405-008-0670-4

[14] Barwad A, Sood S, Gupta N, Rajwanshi A, Panda N, et al (2012) Human papilloma virus associated head and neck cancer: a PCR based study. Diagnostic cytopathology 40: 893–897. 21472871 10.1002/dc.21667

[15] Hoffmann M, Gottschlich S, Görögh T, Lohrey C, Schwarz E, et al (2005) Human papillomaviruses in lymph node neck metastases of head and neck cancers. Acta oto-laryngologica 125: 415–421. 15823814 10.1080/00016480510028528

[16] Desai PC, Jaglal MV, Gopal P, Ghim SJ, Miller DM, et al (2009) Human papillomavirus in metastatic squamous carcinoma from unknown primaries in the head and neck: A retrospective 7 year study. Experimental and Molecular Pathology 87: 94–98. 19393644 10.1016/j.yexmp.2009.04.003

[17] Goldenberg D, Begum S, Westra WH, Khan Z, Sciubba J, et al (2008) Cystic lymph node metastasis in patients with head and neck cancer: An HPV-associated phenomenon. Head & neck 30: 898–903. 18383529 10.1002/hed.20796

[18] Keller LM, Galloway TJ, Holdbrook T, Ruth K, Yang D, et al.. (2013) p16 status, pathologic and clinical characteristics, biomolecular signature, and long term outcomes in unknown primary carcinomas of the head and neck. Head & neck. 10.1002/hed.23514 PMC3972378 24115269

[19] Compton AM, Moore-Medlin T, Herman-Ferdinandez L, Clark C, Caldito GC, et al (2011) Human Papillomavirus in Metastatic Lymph Nodes from Unknown Primary Head and Neck Squamous Cell Carcinoma. Otolaryngology – Head and Neck Surgery 145: 51–57. 21493313 10.1177/0194599811400385

[20] Groppo ER, van Zante A, Yom SS, Eisele DW (2011) Role and Impact of Human Papillomavirus in Cervical Metastasis From Unknown Primary. Laryngoscope 121: S118.

[21] Smeets SJ, Hesselink AT, Speel EJM, Haesevoets A, Snijders PJ, et al (2007) A novel algorithm for reliable detection of human papillomavirus in paraffin embedded head and neck cancer specimen. International journal of cancer 121: 2465–2472. 17680565 10.1002/ijc.22980

[22] Barnes L (2005) Pathology and genetics of head and neck tumours: IARC.

[23] Jacobs MV, Snijders PJ, van den Brule AJ, Helmerhorst TJ, Meijer CJ, et al (1997) A general primer GP5+/GP6(+)-mediated PCR-enzyme immunoassay method for rapid detection of 14 high-risk and 6 low-risk human papillomavirus genotypes in cervical scrapings. Journal of Clinical Microbiology 35: 791–795. 9041439 10.1128/jcm.35.3.791-795.1997 PMC229677

[24] Gronhoj Larsen C, Gyldenlove M, Jensen DH, Therkildsen MH, Kiss K, et al.. (2014) Correlation between human papillomavirus and p16 overexpression in oropharyngeal tumours: a systematic review. Br J Cancer. 10.1038/bjc.2014.42 PMC3960616 24518594

[25] El-Mofty SK, Zhang MQ, Davila RM (2008) Histologic identification of human papillomavirus (HPV)-related squamous cell carcinoma in cervical lymph nodes: a reliable predictor of the site of an occult head and neck primary carcinoma. Head Neck Pathol 2: 163–168. 20614311 10.1007/s12105-008-0066-1 PMC2807564

[26] Sivars L, Näsman A, Tertipis N, Vlastos A, Ramqvist T, et al (2014) Human papillomavirus and p53 expression in cancer of unknown primary in the head and neck region in relation to clinical outcome. Cancer Medicine 3: 376–384. 24510528 10.1002/cam4.199 PMC3987086

[27] Tribius S, Hoffmann AS, Bastrop S, Görögh T, Haag J, et al (2012) HPV status in patients with head and neck of carcinoma of unknown primary site: HPV, tobacco smoking, and outcome. Oral oncology 48: 1178–1184. 22739067 10.1016/j.oraloncology.2012.05.022

[28] Näsman A, Attner P, Hammarstedt L, Du J, Eriksson M, et al (2009) Incidence of human papillomavirus (HPV) positive tonsillar carcinoma in Stockholm, Sweden: An epidemic of viral-induced carcinoma? International Journal of Cancer 125: 362–366. 19330833 10.1002/ijc.24339

[29] Johansen J, Eigtved A, Buchwald C, Theilgaard SA, Hansen HS (2002) Implication of 18F-Fluoro-2-Deoxy-D-Glucose Positron Emission Tomography on Management of Carcinoma of Unknown Primary in the Head and Neck: A Danish Cohort Study. The Laryngoscope 112: 2009–2014. 12439171 10.1097/00005537-200211000-00018

[30] Hoffmann M, Orlamünder A, Sucher J, Gottschlich S, GÖRÖGH T, et al (2006) HPV16 DNA in histologically confirmed tumour-free neck lymph nodes of head and neck cancers. Anticancer research 26: 663–670. 16739336

[31] Zengel P, Assmann G, Mollenhauer M, Jung A, Sotlar K, et al (2012) Cancer of unknown primary originating from oropharyngeal carcinomas are strongly correlated to HPV positivity. Virchows Archiv 461: 283–290. 22855133 10.1007/s00428-012-1290-3

[32] Straetmans JM, Speel EJ, Kremer B (2012) Value of human papillomavirus testing in the diagnostic workup of lymph node metastases from an unknown primary tumor to the neck. Head & neck 34: 1819–1820. 23019180 10.1002/hed.23166

[33] Strojan P, Ferlito A, Medina JE, Woolgar JA, Rinaldo A, et al (2013) Contemporary management of lymph node metastases from an unknown primary to the neck: I. A review of diagnostic approaches. Head & Neck 35: 123–132. 22034046 10.1002/hed.21898

[34] Perkins SM, Spencer CR, Chernock RD, et al (2012) Radiotherapeutic management of cervical lymph node metastases from an unknown primary site. Archives of Otolaryngology–Head & Neck Surgery 138: 656–661. 22801890 10.1001/archoto.2012.1110

[35] Strojan P, Ferlito A, Langendijk JA, Corry J, Woolgar JA, et al (2013) Contemporary management of lymph node metastases from an unknown primary to the neck: II. A review of therapeutic options. Head & Neck 35: 286–293. 22034062 10.1002/hed.21899

[36] Chernock RD, El-Mofty SK, Thorstad WL, Parvin CA, Lewis JS Jr (2009) HPV-related nonkeratinizing squamous cell carcinoma of the oropharynx: utility of microscopic features in predicting patient outcome. Head and neck pathology 3: 186–194. 20596971 10.1007/s12105-009-0126-1 PMC2811624

[37] El-Mofty S, Lu D (2003) Prevalence of human papillomavirus type 16 DNA in squamous cell carcinoma of the palatine tonsil, and not the oral cavity, in young patients: a distinct clinicopathologic and molecular disease entity. The American journal of surgical pathology 27: 1463–1470. 14576481 10.1097/00000478-200311000-00010

[38] Westra WH (2012) The morphologic profile of HPV-related head and neck squamous carcinoma: implications for diagnosis, prognosis, and clinical management. Head and neck pathology 6: 48–54. 22782223 10.1007/s12105-012-0371-6 PMC3394160

